# Calcium dysregulation disrupts mitochondrial homeostasis by interfering AMPK/Drp1 pathway to aggravate plaque progression and instability

**DOI:** 10.7150/thno.112041

**Published:** 2025-06-23

**Authors:** Pingping Hu, Mengmeng Liu, Tongtong Wu, Yiping Zhang, Chenyuan Liu, Langtao Wang, Wanping Zhang, Yumei Que, Jiali You, Weimin Yu, Xiaoyong Tong

**Affiliations:** 1 Chongqing Key Laboratory for Pharmaceutical Metabolism Research, College of Pharmacy, Chongqing Medical University, Chongqing, 400016, China.; 2 School of Pharmaceutical Sciences, Chongqing University, Chongqing, 401331, China.; 3 Department of Cell Biology, Army Medical University, Chongqing, 400038, China.; 4 Tongliang No.1 Middle School, Chongqing, 402560, China.; 5 State Key Laboratory of Frigid Zone Cardiovascular Diseases (SKLFZCD), Harbin Medical University, Harbin, 150000, China.; 6 Chongqing Key Laboratory of New Drug Delivery System, Chongqing, 400038, China.

**Keywords:** SERCA2, mitochondria, AMPK, atherosclerosis, plaque stability

## Abstract

**Rationale**: Inactivation of Cys^674^ (C674) of sarcoplasmic/endoplasmic reticulum calcium ATPase 2 (SERCA2) disrupts intracellular calcium (Ca^2+^) homeostasis and SERCA2 dysfunction has been implicated in the pathogenesis of atherosclerosis and aortic aneurysms. However, the precise role of SERCA2 dysfunction in aortic smooth muscle cells (SMCs) and its contribution to atherosclerosis remains unclear.

**Methods**: We Used heterozygous SERCA2 C674S knock-in (SKI) mice to mimic the partial irreversible oxidation inactivation of C674 thiol under pathological conditions. The whole aorta and aortic root were isolated for immunohistological analysis, RNA sequencing and proteomic analysis. The primary SMCs were collected for cell culture, protein expression and immunofluorescence analysis.

**Results**: Compared with SMCs from WT mice, SKI SMCs demonstrated abnormally activated AMPK/Drp1 pathway adenosine 5'-monophosphate-activated protein kinase (AMPK)/dynamin related protein 1 (Drp1) pathway, and mitochondrial disorders, including increased cytosolic/mitochondrial Ca²⁺ level, oxidative stress, ATP depletion, decreased mitochondrial membrane potential (Δψm), and disrupted mitochondrial dynamics. In SKI SMCs, stimulation of AMPK by metformin or 5-aminoimidazole-4-carboxamide-1-β-D-ribofuranoside (AICAR), or inhibition of Drp1 with mitochondrial division inhibitor 1 (Mdivi-1), restored mitochondrial homeostasis, mitigated excessive matrix metalloproteinase 2 and SMC apoptosis, thereby preserved SMC function.* In vivo administration* of metformin and Mdivi-1 both ameliorated atherosclerosis triggered by SERCA2 dysfunction and particularly enhanced plaque stability.

**Conclusions:** SERCA2 dysfunction accelerates atherosclerotic plaques formation and increases plaque vulnerability by disrupting the AMPK/Drp1 pathway in aortic SMCs, leading to mitochondrial disorders and impairing SMCs function. Targeting of AMPK or Drp1 pharmacologically may offer promising therapeutic avenues for atherosclerosis, particularly in reducing atherosclerotic plaques vulnerability.

## Introduction

Atherosclerosis is a chronic and progressive disease that serves as the principal pathological basis for a variety of cardiovascular diseases 1. The pathogenesis of atherosclerosis is highly complex, involving the synergistic interaction between lipid metabolizers and vascular cell components. Vascular smooth muscle cells (SMCs) are the primary cellular component responsible for the progression and stability of atherosclerotic plaques. SMCs are not the terminally differentiated, which undergo phenotypic switching and can shift from a contractile to a synthetic phenotype in pathological and experimental situation 2. SMCs then migrate from the media layer of artery to the intima and proliferate there, which is crucial not only for vascular media thickening, but also contributing to the formation of fibrous cap and via producing extracellular matrix (ECM) components such as collagen and elastin 3. Additionally, SMCs can also activate proteolytic enzymes, such as matrix metalloproteinases (MMPs) to degrade the fibrous cap and secrete cytokines and chemokines to influence inflammatory environment of the plaques 4. As such, further understanding the mechanisms of atherosclerosis from the perspective of SMCs is critical for the prevention and treatment of the disease.

Calcium ion (Ca^2+^) is the key second messenger in various signaling pathways, and the disruption of Ca^2+^ handling is closely associated with aortic homeostasis and various cardiovascular diseases. Endoplasmic reticulum (ER) is the largest intracellular Ca^2+^ store, and sarcoplasmic/endoplasmic reticulum calcium ATPase 2 (SERCA2) is the sole active transporter on ER membrane that pumps Ca^2+^ from cytoplasm into ER to maintain ER function in cardiovascular system. We previously found that cysteine at position 674 (C674) is a key glutathionylation site that regulates SERCA2 activity, and the irreversible oxidation of SERCA2 C674 is involved in atherosclerosis, aortic aneurysm and pulmonary hypertension 5-7. To simulate the irreversible oxidation of C674 under pathological conditions, cysteine at position 674 was substituted with serine (C674→S674) and the C674S gene mutation knock-in (SERCA2 C674S mutant knock-in, SKI) mouse was constructed 8. Homozygous SKI mice die during the embryonic stage 8, and heterozygous SKI exhibit pronounced atherosclerosis on a genetic background susceptible to atherosclerosis 5. In bone marrow derived macrophages (BMDMs) and endothelial cells (ECs), the substitution of C674 by S674 induces SERCA2 dysfunction, triggers ER stress and inflammation 5. However, the relationship between SERCA2 dysfunction in SMCs and atherosclerosis and the underlying mechanism remains enigmatic.

Mitochondria are not only the main site of oxidative phosphorylation to generate ATP, but also one of the important intracellular calcium stores. Multiple studies have reported that mitochondria and ER are spatially closely connected, and Ca^2+^ dynamics between these two organelles are involved in the regulation of various metabolic pathways 9, 10. Overloading of mitochondrial Ca^2+^ may lead to the opening of mitochondrial permeability transition pore (mPTP), which is always accompanied by an increase in reactive oxygen species (ROS) generation, ultimately resulting in mitochondrial dysfunction and the activation of cell death pathways 11, 12.

In this study, we discovered that SERCA2 dysfunction caused intracellular Ca^2+^ regulation disruption, inducing Ca^2+^ aggregation in cytosol and mitochondria and triggering mitochondrial disorders in SKI aortic SMCs. Moreover, the intracellular Ca^2+^ dysregulation led to compromised AMP-activated protein kinase (AMPK) function and upregulated the expression of dynamin related protein 1 (Drp1). AMPK has been widely accepted to promote Drp1 phosphorylation, while phosphorylated Drp1 translocates from the cytoplasm to the mitochondrial outer membrane to initiate mitochondrial fission 13. However, the relationship between AMPK/Drp1 pathway and mitochondrial homeostasis are not clear. We further identified that the intervention targeting AMPK and Drp1 can both reverse SERCA2 dysfuntion induced mitochondrial disorders in SMCs and ameliorate atherosclerosis in SKI mice. Notably, *in vivo* intervention targeting the AMPK/Drp1 pathway was found to effectively promote plaque stability with increased thickness of the fibrous cap. Our findings may pave a new avenue for developing effective medicine to treat atherosclerosis as well as plaque vulnerability.

## Methods

### Animal experiments

All animal care and study protocols are complied with the guidelines of the ethical use of animals and are approved by the Laboratory Animal Welfare and Ethics Committee of Chongqing Medical University (IACUC No.: CQU-IACUC-RE-2024101). Mice are bred and housed in the animal room at Chongqing University under specific pathogen-free conditions. Mice were kept in open polypropylene cages with clean chip bedding. The animal room is maintained at a controlled temperature (22 ± 3 °C) and a 12 h cycle of light and dark. Five mice in each cage are free to drink water and fed on a regular diet (0.3-0.8% sodium, 1.0-1.8% calcium, 0.6-1.2% phosphorus, Beijing Keao Xieli Feed Corporation, China) unless otherwise indicated. To obtain tissues, all mice are killed by intraperitoneal injection instantly with avertin (2,2,2-tribromoethanol, Sigma-Aldrich, Cat# T48402) at a dose of 300 mg/kg.

All mice used in this study are of C57BL/6J background (The Jackson Laboratory, Bar Harbour, Maine, USA). A mutated 14.4 kb SERCA exon 14 containing the Cys-674 to Ser-674 codon (TGT→TCC) was inserted into the endogenous SERCA2 gene locus, and SERCA2 C674S knock in (SKI) mouse was constructed as previously described 8, 14. Since the homozygous SKI mice is embryonal lethal, only heterozygous SKI mice with 50% of C674 and 50% of S674 were used in this study. The littermate wild-type (WT) mice without S674 were used as controls. For atherosclerosis studies, SKI mice were mated with low-density lipoprotein receptor deficiency (*ldlr*^-/-^, The Jackson Laboratory) mice to obtain SKI *ldlr^-/-^* mice, and the littermate *ldlr*^-/-^ mice were served as controls.

### High-fat feeding induces atherosclerosis

WT and SKI mice of *ldlr*^-/-^ background at 8 weeks old (18-22 g) were fed with a high-fat diet (2% cholesterol, 16.4% lard and 3.6% vegetable oil, Beijing Keao Xieli Feed Corporation) for 8 weeks. At the same time, AMPK agonist-metformin (Met, MCE, Cat# HY-17471A/CS-1851, 260 mg·kg^-1^·day^-1^, dissolved in 200 μL ddH_2_O) or its control solvent was given by gavage for 8 weeks to identify the contribution of deactivated AMPK in SKI mice. To investigate the effect of aberrant mitochondrial fission on SERCA2 dysfunction directed atherosclerosis, mitochondrial division inhibitor 1 (Mdivi-1, 50 mg/kg, Beyotime, Cat# SC8028, dissolved in 10% DMSO supplemented with 40% PEG300, 5% Tween-80 and 45% Saline) or its control solvent was given by intraperitoneal injection at 9:00-10:00 in the morning twice a week with supplementary of high-fat diet for 8 weeks. Changes in body weight of mice were recorded weekly. Mice were sacrificed and perfused with ice-cold PBS, and then the connective tissue was removed to expose the whole aorta, and its collateral branches and digital images were acquired for atherosclerosis analysis using an Olympus microscope (model SZX16).

### RNA sequencing and bioinformatics

Aorta of 4-month male from WT and SKI (*ldlr*^-/-^) mice were homogenized in Trizol for RNA isolation using the Direct-zol™ RNA MiniPrep Kit (Zymo Research, Irvine, CA). RNA sequencing was administrated in Novogene Bioinformatic Technology Co. Ltd (Beijing, China). Differentially expressed genes between WT and SKI mice were analyzed using DESeq R package. Gene set enrichment analysis was performed on differentially expressed genes (DEGs, altered >1.5 fold) 15, 16 to test for enrichment of specific ontologies, and the Kyoto Encyclopedia of Genes and Genomes (KEGG, http://www.genome.jp/kegg/) was used to perform pathway analysis.

### Quantitative proteomic analysis

Aorta of 4-month-old male WT and SKI (*ldlr*^-/-^) mice were grinded by liquid nitrogen, incubated with four volumes of lysis buffer (8 M urea, 1% Protease Inhibitor Cocktail) and followed by sonication to collect the total proteins. 100 μg protein per sample was then subjected to tryptic digestion after reduction (10 mM DTT) and alkylation (55 mM iodoacetamide). The digested peptides from WT and SKI groups were separately labeled with distinct TMT reagents, desalted and fractionated by high-pH reverse-phase HPLC and analyzed by liquid chromatography-tandem mass spectrometry (LC-MS/MS) on an Orbitrap Fusion Lumos instrument operated in data-dependent acquisition (DDA) mode. Raw data were processed using Proteome Discoverer 2.4, searching against the UniProt Mus musculus database. Protein quantification was performed based on TMT reporter ion intensities, with normalization across all channels. Differentially expressed proteins (DEPs) were filtered using thresholds of |fold change| > 1.5 and adjusted p-value < 0.05 (Benjamini-Hochberg correction). To further identify the effect of SERCA2 dysfunction on molecular interaction networks, KEGG pathway annotation was used, and the result is mapped using KEGG online service tools. DEPs identified from the proteomic analysis were subjected to KEGG functional enrichment by comparing against the background of all detected proteins. Then, a two-tailed Fisher's exact test was employed to test the enrichment of the differentially expressed protein against all identified proteins. The enriched pathway with a corrected p-value < 0.05 was considered significant.

### Measurement of plasma cholesterol and triglycerides

Mice were sacrificed and blood was taken from the right atrium into heparin-containing tubes for lipid measurements. Plasma was prepared via centrifugation at 2,000 rpm for 10 min at 4 °C and stored at -80 °C until usage. Measurements were carried out using the Infinity Cholesterol and Triglycerides measurement kits (Thermo Scientific, USA, Cat# TR13421, Cat# TR22421), based on the absorbance of samples normalized to that of a known concentration of a calibrator provided in the kits.

### Histology

Tissues were carefully dissected and fixed in 4% paraformaldehyde overnight, followed by 30% sucrose solution for 24 h before being embedded in optimal cutting temperature compound (OCT compound) for preparation of serial frozen sections. 7 μm-thick sections of aortic root from each mouse were stained individually with hematoxylin/eosin (HE) and Oil red O for morphological analysis, lipid deposition assessment and collagen deposition.

### Immunofluorescence

Frozen sections of aortic root were incubated with primary antibody against 8-Hydroxy-2'-deoxyguanosine (8-OHdG, Bioss, Cat# bs-1278R, dilution 1:300) or caspase 3 (Proteintech, Cat# 19677-1-AP,32 kDa, dilution 1:200) at 4°C overnight, then washed with PBS, and incubated with secondary antibody Cy3-conjugated goat anti-mouse IgG (Proteintech, Cat# SA00009-1, dilution 1:300). Mouse IgG (Santa Cruz Biotechnology, Cat# sc-2025, dilution 1:200) was used as a negative control. Fluorescence signals were recorded by fluorescence microscopy (Leica DM6).

### Aortic SMC culture

Aortic SMCs were isolated from 4-6 weeks old WT and/or SKI mice of C57BL/6J background and cultured in DMEM supplemented with 10% FBS (ExCell Bio, Cat# FSP500), 100 U/mL penicillin and 100 μg/mL streptomycin at 37 °C in a humidified atmosphere containing 5% CO_2_ 6. SMC phenotype was confirmed by α-SMA immunostaining, and SMCs from passages 3 to 8 were used. In some experiments, SMCs were treated with Mdivi-1 (10 μM), metformin (1 mM), AICAR (200 μM, Selleck, Cat# S1802) or Gingerol (40 μM, MCE, Cat# HY-14615), before being collected for western blot analysis or cell function studies. 10% DMSO served as a solvent control.

### Cell proliferation assay

5×10^3^ SMCs /well were seeded in 96-well plate in DMEM supplemented with 0.2% FBS overnight, and fresh medium with 10% FBS was used to stimulate cell proliferation. Cell number was determined 48 h later using a tetrazolium-based non-radioactive proliferation assay kit (Quick Cell Proliferation Assay Kit II, BioVision, Cat# K301-500) according to the manufacturer's protocol. In some experiments, Mdivi-1, metformin or AICAR was added to the culture media for 48 h, and 10% DMSO served as a solvent control.

### Wounded monolayer migration assay

1.0×10^6^ cells/well in 12-well plate were seeded in DMEM with 0.2% FBS overnight. A pipet tip was then used to scratch wounds in SMC monolayer, and the cells were treated with 10% FBS DMEM immediately to stimulate cell migration. Photographs at three fixed locations along the scratch were taken at 0 h and 6 h with a light microscope and analyzed with NIH Image J software.

### Bone marrow derived macrophage (BMDM) adhesion assay

Bone marrow cells were harvested from WT mice and cultured and differentiated to macrophage in high glucose DMEM with 10% FBS and supplemented with 20% L929-conditioned media as previously reported 5. For macrophage adhesion assay, SMCs were plated in 12-well plates (10^6^ cells/well) and cultured in 0.2% FBS DMEM overnight. 5×10^5^ cells/well of the matured bone marrow derived macrophages (BMDMs) were added to SMCs and incubated for 1 h, and the media was then removed, and the cells were washed 3 times with PBS to remove all unbound macrophages. In some experiments, SKI SMCs were pretreated with metformin, AICAR or Mdivi-1 for 24 h before macrophage adhesion assay. As reported previously 5, 6, four images of macrophage adhesion to SMCs in each well were taken and the number of bound macrophages was counted per image area.

### Measurement of cytosolic, ER and mitochondrial Ca^2+^

Aortic SMCs were seeded on glass coverslips overnight in a 24-well plate. According to the manufacture's procedure, the cells were loaded with Fluo-4 AM (Solarbio, Cat# F8500) for cytosolic Ca^2+^, BBcellProbe® C93 (Cat# BB-481159, Bestbio, China) for ER Ca^2+^ or Rhod 2 (Abcam, Cat# 145037-81-6) for mitochondrial Ca^2+^ labelling in HBSS (Hyclone, Cat# SH30030.02) in the dark at 37 °C for 30 min, then were retained for 40 min after adding 2-fold volume of HBSS containing 10% FBS. SMCs were washed 5 times with HEPES buffer, and incubated in the dark at 37 °C for 10 min. Fluorescence pictures were collected by fluorescence microscopy (Leica DM6, Germany) and the average fluorescence intensity was analyzed by Las X software (Leica, Germany). Briefly, open the image that needed to be analyzed in Las X software and select the specific area for statistics. The software then processes the data and displays the average fluorescence intensity.

### Mitochondrial membrane potential assessment

Aortic SMCs were incubated with an equal volume of JC-1 staining working solution (Cat# M8650, Solarbio, China) at 37 °C for 20 min in the dark. The cells were then collected by centrifuged (600 × g, 4 °C, 3 min) and washed twice with JC-1 staining buffer to remove unbound dye. Finally, the stained cells were resuspended in the staining buffer and analyzed using flow cytometry or fluorescence microscopy (Beckman CytoFLEX S, China).

### Analysis of mitochondrial network structure

Aortic SMCs were seeded on glass coverslips overnight in a 24-well plate. Cells were incubated with 50 nM Mito-tracker (Beyotime, Cat# C1049B) in HBSS (Hyclone, Cat# SH30030.02) containing 0.02% pluronic F12 in the dark at 37 °C for 30 min and then washed 3 times with PBS. Fluorescence images were collected (Ex: λ579 nm) by fluorescence microscopy (Leica DM6, Germany). The number of mitochondrial individuals, branch length, and mitochondrial area were analyzed using plug-in NINA software. Mitochondrial fragmentation index (MFI) was represented as the ratio of individuals/ mitochondrial area.

### Mitochondrial ATP measurements

SMCs were seeded in a 24-well plate and cultured in the medium containing s-GQDs (50 μg/mL) for another 12 h more. SMCs were washed 3 times with PBS and finally imaged under a confocal microscope (Leica DM6, Germany). The average fluorescence intensity was analyzed by Las X software (Leica, Germany) as mentioned above.

### Real-time quantitative PCR

1×10^6^ SMCs were cultured in resting Medium with 0.2% FBS for 12 h. The total RNA was isolated using TRIzol reagent (Invitrogen, Cat# 15596026) and retro-transcribed to cDNA using cDNA PCR kit (Takara Bio Inc., Cat# RR037Q). Real-time quantitative PCR was performed using SYBR-Green-based detection (Takara Bio Inc., Cat# RR420L). β-actin was used as internal control.

### Western blot

SMCs rested in 0.2% FBS DMEM overnight were lysed in radio immunoprecipitation assay (RIPA) lysis buffer (Enogene, Cat# E1WP106). Proteins were separated by SDS-PAGE electrophoresis, transferred to PVDF membrane, and immunoblotted with specific antibodies overnight at 4 °C against phospho-AMPK alpha (Thr172) (p-AMPK, Affinity Biosciences Cat# AF3423, 62 kDa, dilution 1:1000), AMPK (Proteintech, Cat# 18167-1-AP, 62 kDa, dilution 1:1000), ATP synthase subunit 6 (ATP6, Proteintech, Cat# 15280-1-AP, 31 kDa, dilution 1:1000), NADH dehydrogenase subunit 1 (ND1, Proteintech, Cat# 19703-1-AP, 28 kDa, dilution 1:2000), mitochondrial cytochrome-c oxidase subunit II (mtCOX II, Proteintech, Cat# 55070-1-AP, 23 kDa, dilution 1:1000), mtCOX IV (Proteintech, Cat# 11242-1-AP, 17 kDa, dilution 1:1000), peroxisome proliferator-activated receptor-gamma coactivator 1 alpha (PGC1α, Proteintech, Cat# 66369-1-Ig, 100 kDa, dilution 1:1000), mitochondrial transcription factor A (TFAM, Proteintech, Cat# 22586-1-AP, 25 kDa, dilution 1:1000), fission 1 (Fis1, Proteintech, Cat# 10956-1-AP, 17 kDa, dilution 1:1000), mitochondrial elongation factors 51 (Mid51, Proteintech, Cat# 20164-1-AP, 51 kDa, dilution 1:1000), mitofusin 2 (MFN2, Proteintech, Cat# 12186-1-AP, 86 kDa, dilution 1:1000), optic atrophy 1 (OPA1, Proteintech, Cat# 27733-1-AP, 100 kDa, dilution 1:1000), MMP2 (Proteintech, Cat# 10373-2-AP, 72 kDa, dilution 1:750), caspase 3 (Proteintech, Cat# 19677-1-AP,32 kDa, dilution 1:1000), cleaved caspase 3 (Cl-Cas3, Proteintech, Cat# 25128-1-AP, 17 kDa, dilution 1:1000) or β-actin (Proteintech, Cat# 20536-1-AP, 45 kDa, dilution 1:5000), followed by incubation with HRP-conjugated goat anti-rabbit secondary antibody (Sino Biological Inc., Cat# SSA003, dilution 1:15000) 1 h at room temperature. Proteins were visualized with ChemiDoc™ Touch System (Bio-Rad, USA). Band density was quantified by NIH Image J software (https://imagej.net/) and normalized to β-actin.

### Data and statistical analysis

For data analysis, the assessors were blinded to animal code. That is, the animal code was assigned by one person, while data analysis was done by others without knowing the animal code, then data were regrouped based on animal code. Group size is the number of independent repeats indicated in figure legend, and statistical analysis was undertaken only using these independent repeats with n ≥ 5. Sample sizes in each group subjected to statistical analysis were determined based on our previous studies, preliminary results, and power analysis. The mean values of the control group were normalized to 1. In the quantitative figures of western blot, Y axis shows the ratio of the values in experimental group to that in control group to avoid the larger variation among different experiments. Statistical analysis was performed with GraphPad Prism 9.0 (http://www.graphpad.com). Unpaired t test was used to analyze data from two different groups. Differences between WT and SKI mice with or without Met/AICAR or Mdivi-1 treatment were analyzed by one-way ANOVA with Tukey's multiple comparisons test. To determine whether groups differed, the level of probability was set at *p* < 0.05 for statistical significance.

## Results

### SERCA2 dysfunction inhibits mitochondrial respiration and interferes SMCs function

**Figure 1:**
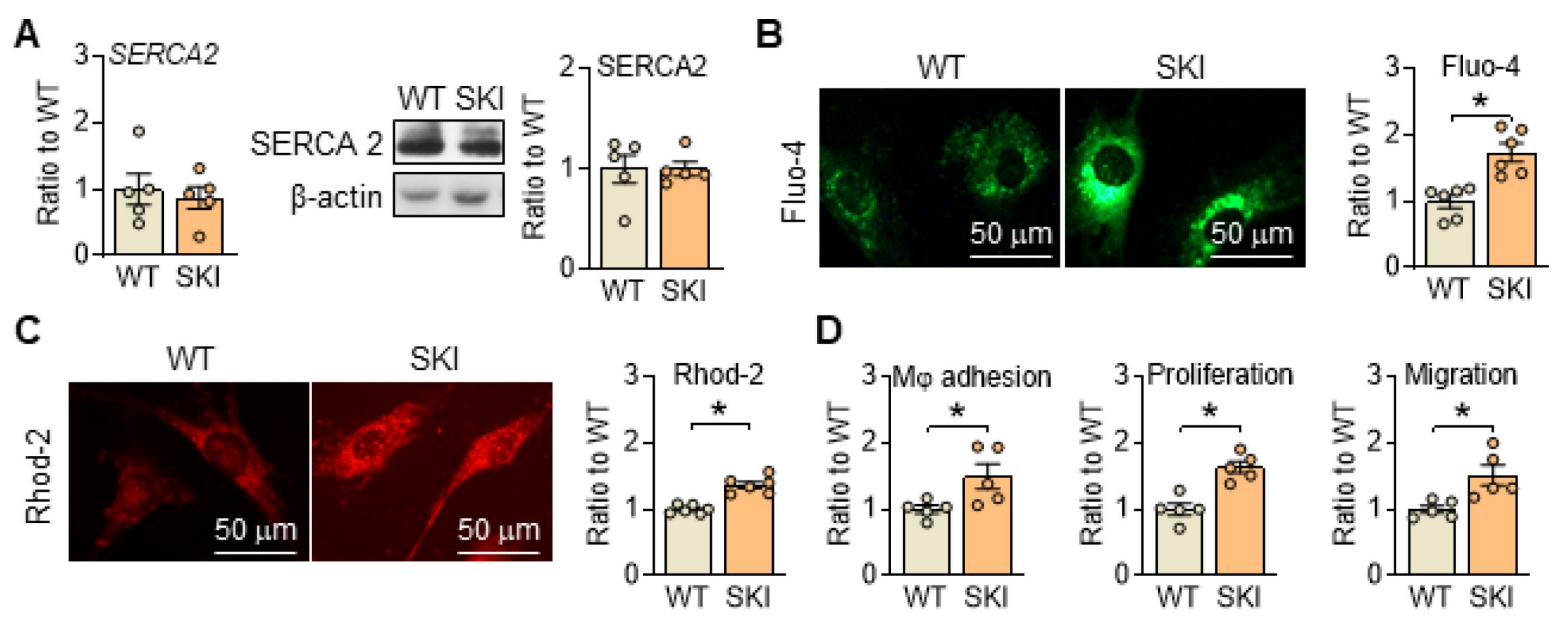
SERCA2 dysfunction disrupts intracellular Ca^2+^ homeostasis and SMC functions, oxidative phosphorylation and mitochondrial biogenesis in SKI SMCs. (A) mRNA and protein levels of SERCA2 in SMCs from WT and SKI mice. Representative images of intracellular Ca^2+^ detected by Fluo-4 (B) and Rhod-2 (C), and the quantification of density in the graph. (D) The quantitative analysis of macrophage (Mφ) adhesion, SMCs proliferation and migration. *p < 0.05 SKI *vs*. WT, n = 5-6 in each group. Data represent the mean of experiments ± SEM, unpaired Student *t*-test.

Disrupted Ca^2+^ homeostasis is recognized as a major contributor to atherogenesis-associated processes 17, 18. To mimic the Ca^2+^ dysregulation caused by irreversible oxidation deactivation of C674 in SERCA2 under pathological conditions, SKI mouse line was constructed. As shown in Figure 1A, the substitution of C674 with S674 had no obvious effect on the expression of SERCA2 (mRNA and/or protein) but induced significant elevated cytosol and mitochondrial Ca^2+^ levels detected by fluorescent intensity of Fluo-4 and Rhod 2 in SMCs (Figure 1B-C). Correspondingly, SERCA2 dysfunction promoted the adhesion of macrophages to SMCs, and enhanced SMC proliferation and migration (Figure 1D). These data indicated that C674 substitution caused SERCA2 dysfunction, interfered intracellular Ca^2+^ homeostasis and thus led to impaired SMC functions, which is consistent with our previous studies 5, 6.

### SERCA2 dysfunction disrupts mitochondrial homeostasis

**Figure 2:**
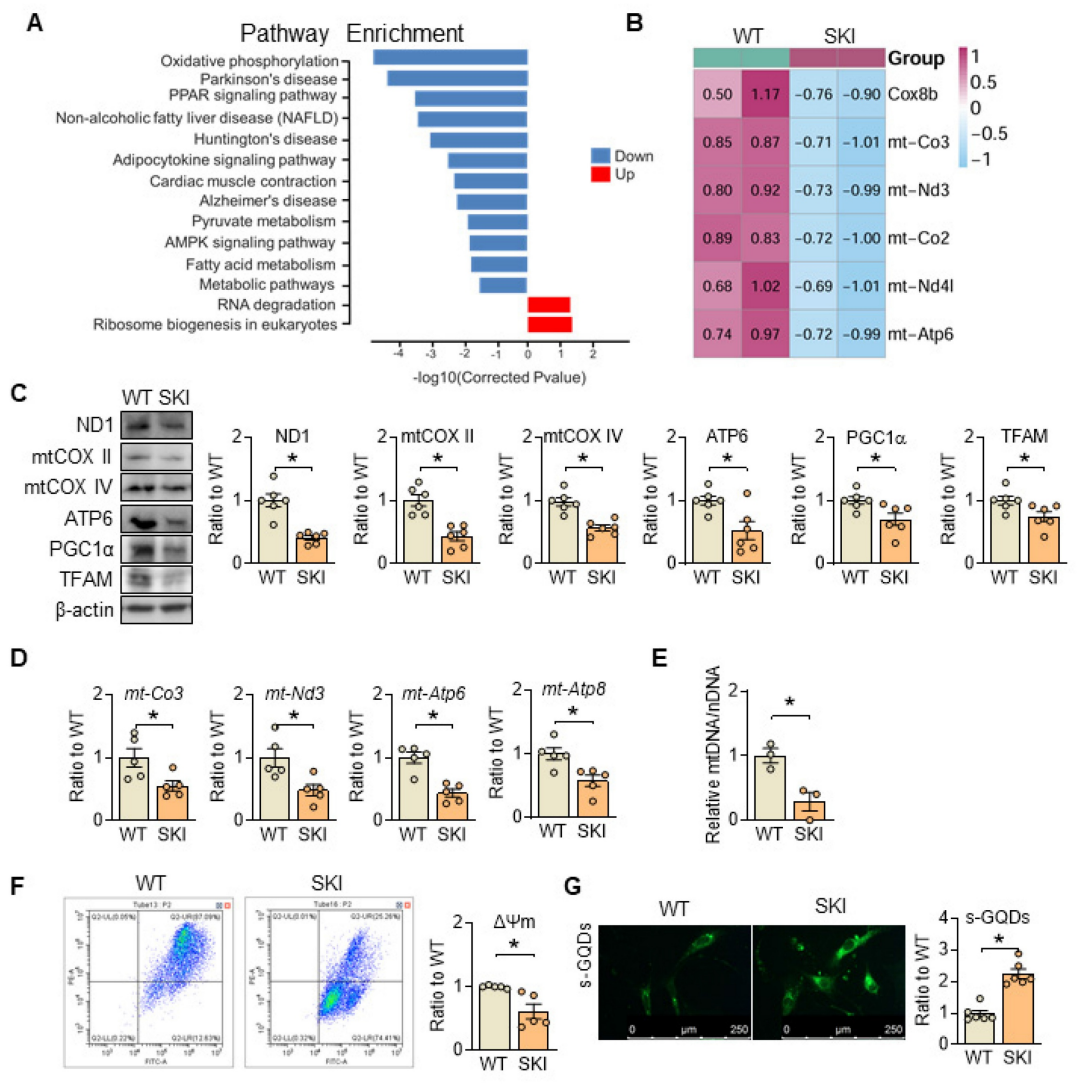
SERCA2 dysfunction causes mitochondrial impairment. (A) The main signaling pathways from the *Kyoto Encyclopedia of Genes and Genomes* pathway enrichment analysis in aorta samples of WT and SKI mice under *ldlr*^-/-^ background. (B) Heatmap for all the DEGs in oxidative phosphorylation pathway. (C) Representative western blots from WT and SKI SMCs and quantification of band intensities in graph. (D) mRNA levels of *mt-CO3*, *mt-Nd3*, *mt-Atp6* and* mt-Atp8* in SMCs from WT and SKI mice. (E) Mitochondrial copy numbers of WT and SKI SMCs. (F) Mitochondrial membrane potential (Δψm) assessment of WT and SKI SMCs. (G) Representative fluorescent images and associated quantification of WT and SKI SMCs incubated with s-GQDs. *p < 0.05 SKI *vs*. WT, n = 5-6 in each group. Data represent the mean of experiments±SEM, unpaired Student *t*-test.

Pathway enrichment analysis from RNA sequencing of wild-type (WT) and SKI aorta indicates that SERCA2 dysfunction impairs oxidative phosphorylation (Figure 2A). The mRNA and protein levels of genes responsible for the electron transport chain (ETC) are all downregulated (Figure 2B-D), accompanied with the downregulation of proteins related with mitobiogenesis (PGC1α and TFAM) (Figure 2C) and decreased mitochondrial copy number (Figure 2E), while the mitochondrial membrane potential was also significantly reduced in SKI SMCs (Figure 2F). Moreover, using s-GQDs immunofluorescence staining to detect ATP production, whose fluorescence signal is negatively correlated with ATP content 19, 20, an obvious decreased ATP production was observed in SKI aortic SMCs (Figure 2G).

**Figure 3:**
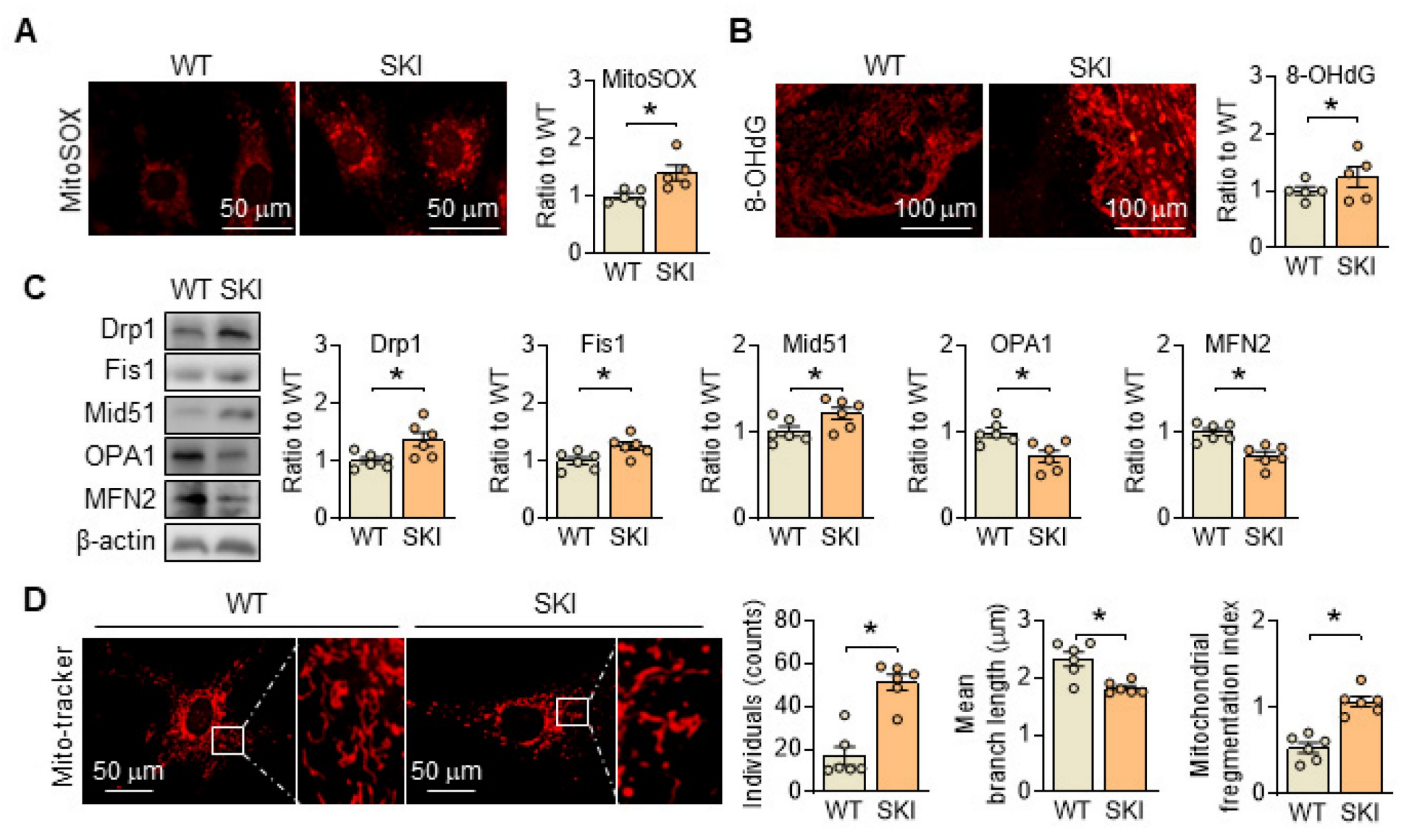
SERCA2 dysfunction triggers oxidation damage and promotes mitochondrial fission. (A) Representative fluorescent images and associated quantification of WT and SKI SMCs incubated with MitoSOX. (B) Representative immunofluorescence against 8-OHdG in the atherosclerotic lesions of aortic root from WT and SKI mice and associated quantification of the fluorescent intensity in the graphs. (C) Representative western blots of proteins related with mitochondrial fission and fusion (Drp1, Fis1, Mid51, OPA1 and MFN2) from the WT and SKI SMCs and quantification of band intensities in graph. (D) Representative images and associated quantification of mitochondrial morphology in WT and SKI SMCs incubated with Mito-tracker. *p < 0.05 SKI *vs*. WT, n = 5-6 in each group. Data represent the mean of experiments±SEM, unpaired Student *t*-test.

Besides producing ATP, mitochondria are also the primary source of reactive oxygen species (ROS) in the cell, and their generation is closely linked to ETC and ATP production. In SKI SMCs, the positive MitoSOX staining, which is a selective mitochondrial superoxide indicator, increased significantly, indicated an elevated ROS content (Figure 3A). Correspondingly, 8-OHdG positive staining was also significantly increased in the atherosclerotic lesions of aortic root from SKI mice, suggested that SERCA2 dysfunction resulted in an elevated content of mitochondrial ROS and severe oxidative damage (Figure 3B). Moreover, in SKI SMCs, we found that the genes responsible for mitochondrial fission, including Drp1 and Fis1, were upregulated, while genes responsible for mitochondrial fusion, such as Mfn2 and Opa1, were downregulated (Figure 3C). Mito-tracker staining indicates an increase in mitochondrial fission in SKI SMCs (Figure 3D). All of these data suggested that SERCA2 dysfunction disrupted mitochondrial and SMC functions by interfering intracellular Ca^2+^ handling.

### SERCA2 dysfunction impairs AMPK signaling pathway in aortic SMCs

**Figure 4:**
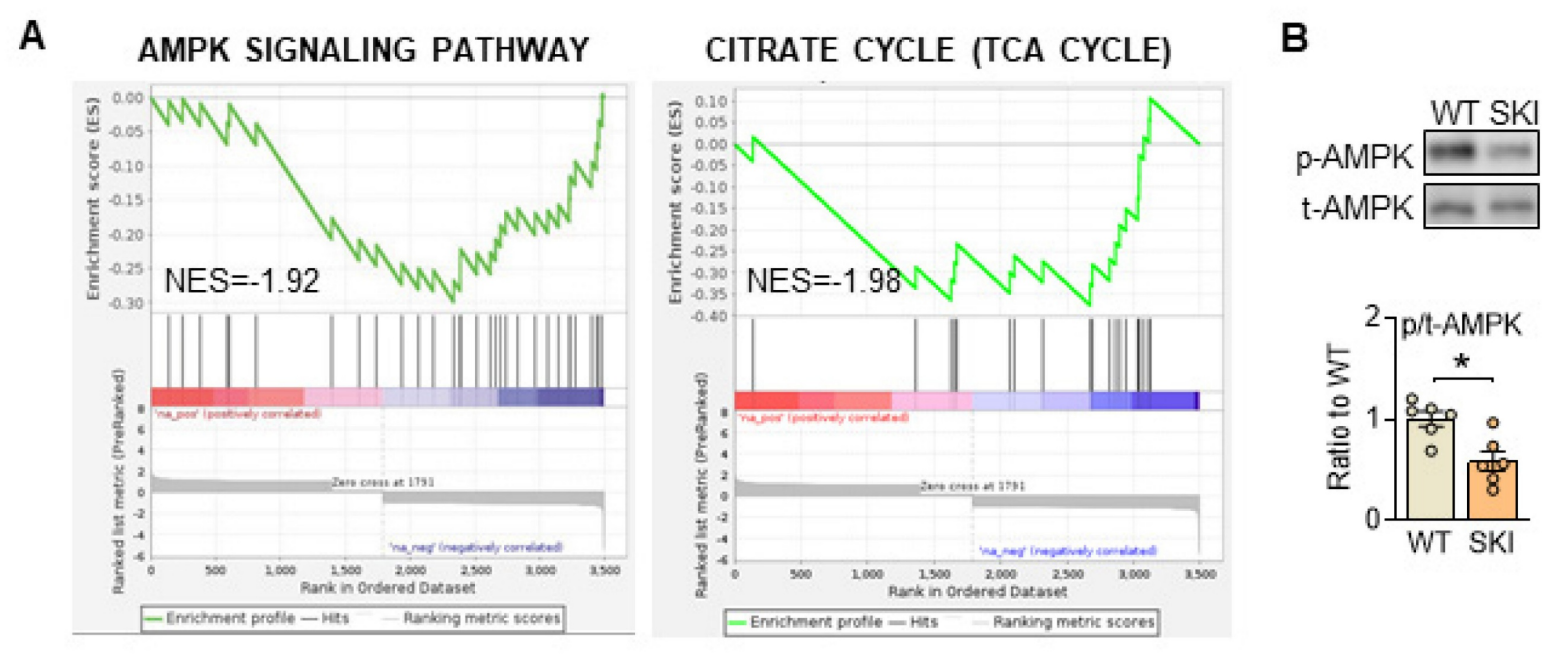
SERCA2 dysfunction suppresses AMPK pathway in SMCs. (A) Significant enrichment of AMPK and TCA pathway expression signatures. (B) Representative western blots of phosphorylated and total AMPK in WT and SKI SMCs. n = 6. *p < 0.05 SKI vs. WT. Data represent the mean of experiments±SEM, unpaired Student *t*-test.

To further investigate the mechanism that SERCA2 dysfunction directed mitochondrial abnormities, quantitative proteomic analysis of aorta from WT and SKI (*ldlr*^-/-^) mice was also administrated. As shown in supplementary Figure 1, bioinformatics analysis revealed that among the 2,736 differentially expressed proteins detected, 124 were upregulated and 53 were downregulated in SKI mice, while AMPK and TCA cycle signaling pathways were both significantly downregulated in SKI mice from the KEGG enrichment analysis of these differential proteins (Figure 4A). Additionally, AMPK pathway was also significantly downregulated from RNA sequencing of aorta (Figure 2A). AMPK is wildly known as the energy sensor and critical regulator for mitochondria homeostasis, which can be activated through the phosphorylation of a specific threonine residue (Thr-172) and thus modulate energetic metabolism in response to Ca^2+^ flux or energy-deprivation 21, 22. As shown in Figure 4B, SERCA2 dysfunction significantly reduced the protein expression levels of phosphorylation of AMPK, indicated the AMPK signaling pathway defects were involved in SKI SMCs.

### Stimulation of AMPK by metformin and AICAR both improve mitochondrial function in SKI aortic SMCs

**Figure 5:**
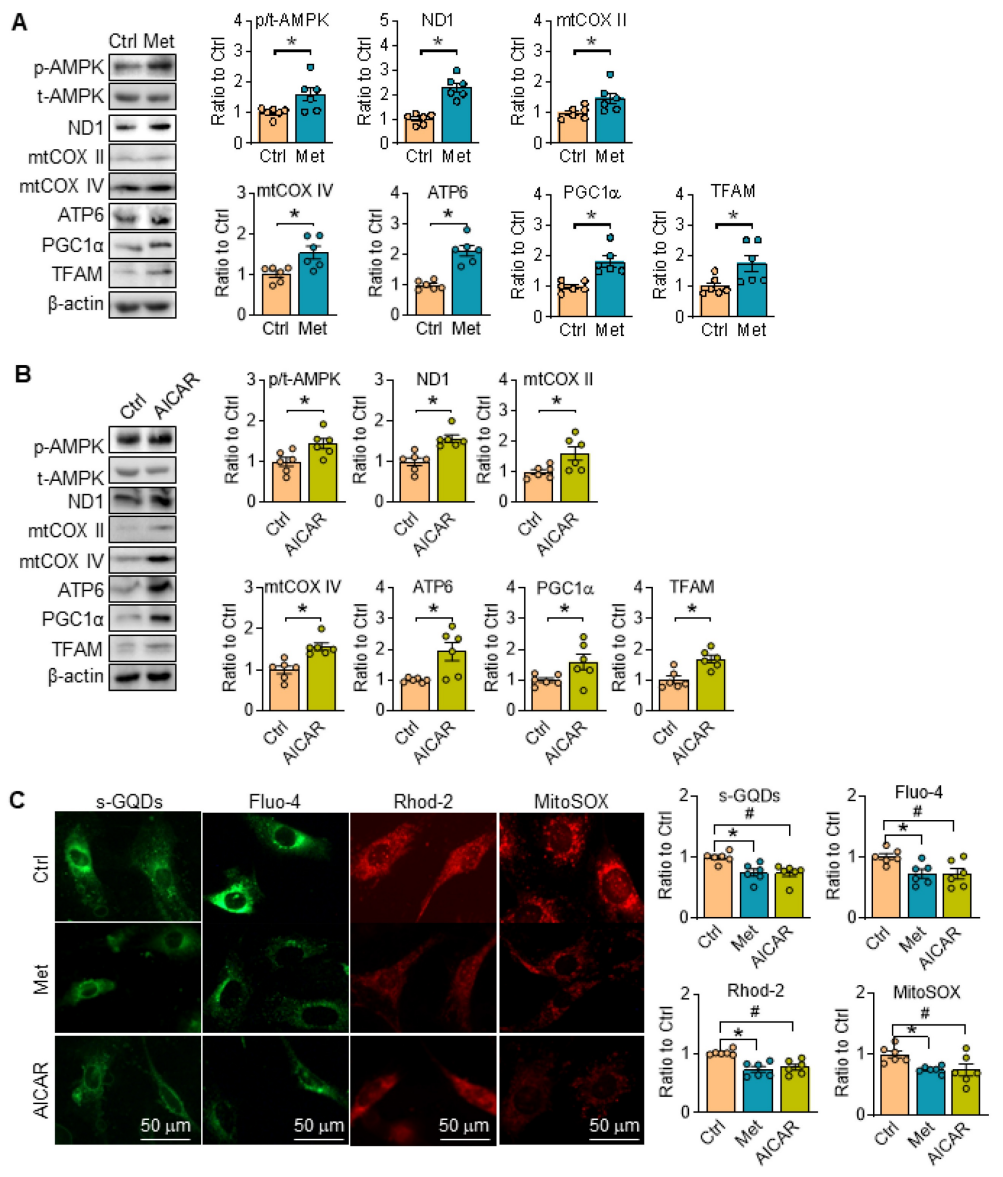
Activation of AMPK restores mitochondrial function in SKI SMCs. Representative western blots of p-AMPK and proteins related with oxidative phosphorylation (ND1, mtCOX II, mtCOX IV and ATP6) and mitochondrial biogenesis (PGC1α and TFAM) in SKI SMCs with or without the administration of metformin (Met, A) and AICAR (B) or the solvent control (Ctrl) in SKI SMCs and quantification of band intensities in graph. n=6 in each group. *p < 0.05 Met or AICAR *vs*. Ctrl, Data represents the mean of experiments±SEM, unpaired Student *t*-test. (C) Representative images and associated quantification of the fluorescence of s-GQDs, Fluo-4, Rhod-2 and MitoSOX in SKI SMCs with the treatment of metformin (Met) and AICAR or the solvent control (Ctrl). n = 6. *p < 0.05 Met vs. Ctrl; #*p* <0.05 AICAR vs. Ctrl. Data represents the mean of experiments±SEM, One-way ANOVA with Tukey's multiple comparisons test.

Reportedly, metformin is a widely used agonist of AMPK through multiple pathway 23, 24, while 5-aminoimidazole-4-carboxamide-1-b-d-ribofuranoside (AICAR) is an AMP analogue, and can bind AMPK subunits to activate AMPK 25. Herein, metformin and AICAR were utilized in SKI SMCs, and they both can promote the phosphorylation of AMPK, and reverse the expression of proteins related with ETC (ND1, mtCOX II, mtCOX IV and ATP6), PGC1α and TFAM in SKI SMCs (Figure 5A-B). Simultaneously, the fluorescent intensity of sGQDs, Fluo-4, Rhod-2 and MitoSOX in SKI SMCs were all reversed with metformin or AICAR administration in SKI SMCs (Figure 5C).

### AMPK activation restores mitochondrial dynamics and promotes SKI SMCs functions

**Figure 6:**
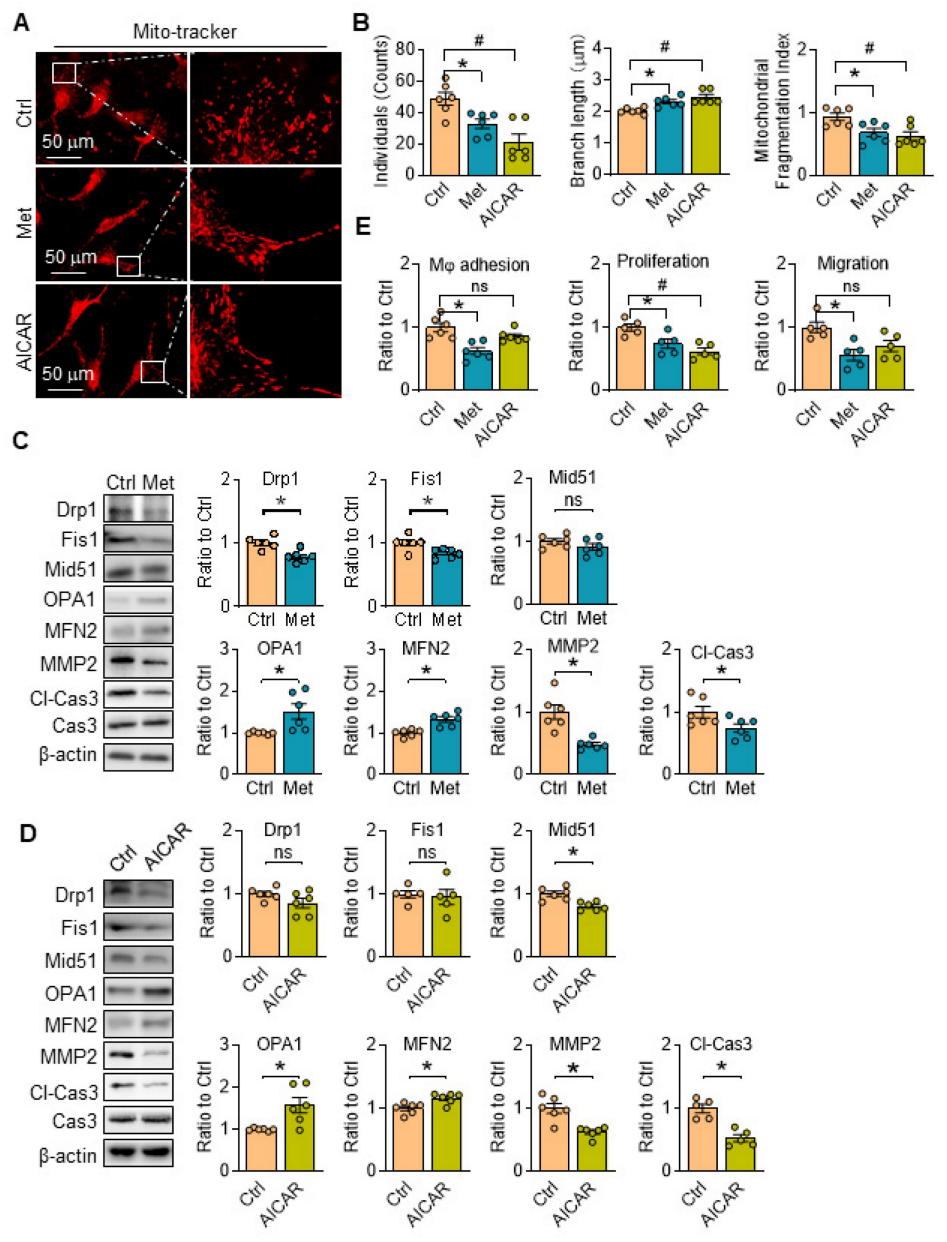
AMPK stimulation ameliorates SERCA2 dysfunction induced mitochondrial fragmentation and restore SMC function. Representative images (A) and associated quantification (B) of mitochondrial morphology in SKI SMCs with or without treatment of metformin or AICAR. n = 6. *p < 0.05 Met vs. Ctrl; #*p* < 0.05 AICAR vs. Ctrl. Data represents the mean of experiments±SEM. One-way ANOVA with Tukey's multiple comparisons test. Representative western blots of proteins in SKI SMCs with or without the administration of metformin (C) or AICAR (D) and quantification of band intensities in graph. *p < 0.05 Met or AICAR *vs*. Ctrl, n = 5-6 in each group. Data represents the mean of experiments±SEM, unpaired Student *t*-test. (E) The quantitative analysis macrophage adhesion, proliferation and migration of SKI SMCs with or without treatment of Met or AICAR. n = 5-6. *p < 0.05 Met vs. Ctrl; #*p* < 0.05 AICAR vs. Ctrl. Data represent the mean of experiments±SEM. One-way ANOVA with Tukey's multiple comparisons test.

There are no related reports about the functions of metformin and/or AICAR on mitochondria morphology in aortic SMCs, the changes of which was then investigated with AMPK activation. We found mitochondrial individuals, branch length and fragmentation of SKI aortic SMCs were all evidently reduced treated with metformin, accompanied with downregulated fission-associated proteins (Drp1 and Fis1) and upregulated fusion-associated proteins (OPA1 and MFN2) (Figure 6A-C). It's worth noting that metformin intervention reduced the expression of MMP2 and Cl-Cas3, which are closely associated with plaques rupture 26, and both of them are upregulated in SKI aortic SMCs in our previous work 6, 27. Similar results on mitochondrial dynamics were observed when the cells were treated with AICAR with the exception of no changes in the expression of Drp1, Fis1 and MMP2 (Figure 6A, B and D). Of note, the macrophage adhesion, proliferation and migration of SKI SMCs were also markedly suppressed with the metformin administration, while AICAR treatment can also reduce macrophage adhesion to SMCs (Figure 6E). All of these results revealed that AMPK stimulation with metformin and AICAR can both restore mitochondrion homeostasis and improve SMC functions.

### AMPK stimulation by metformin ameliorates SERCA2 dysfunction induced atherosclerosis

**Figure 7:**
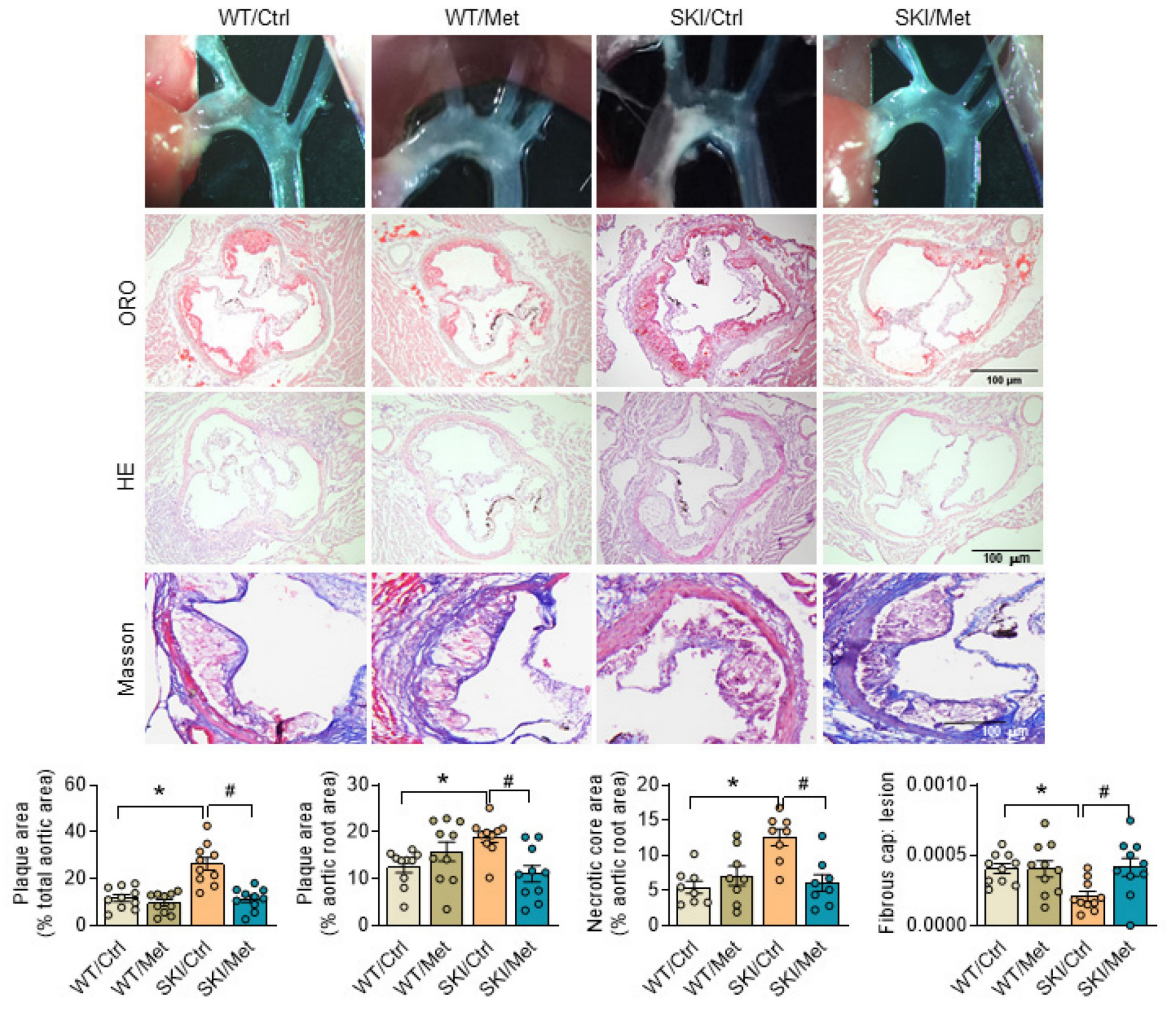
Activation of AMPK by metformin relives atherosclerosis in SKI mice. Representative images of aortic arch, lesions stained with oil red O, necrosis stained with H&E and collagen deposition stained with Masson in aortic root (up panel) and the quantification in the graph (down panel). n = 10. *p < 0.05 SKI/Ctrl vs. WT/Ctrl; #*p* < 0.05 SKI/Met vs. SKI/Ctrl. Data represent the mean of experiments±SEM, One-way ANOVA with Tukey's multiple comparisons test.

Considering the excellent agonistic effect of metformin on mitochondrial homeostasis and SMC function, metformin was then given to SKI and WT mice by daily administration (gavage) for 8 weeks, which were placed on a western diet simultaneously. Aortas were then harvested and stained with oil red O to visualize atherosclerotic plaques. Both the pictures of aortic arch and oil red O staining of aortic root showed that SERCA2 dysfunction accelerated the progression of atherosclerosis. As shown in Figure 7, SKI mice developed a 2.25-fold and 1.5-fold increase of plaques in the aorta arch and aortic root, respectively, while metformin treatment resulted in an obvious decrease of lesions in both of the aorta arch and aortic root. H&E staining also showed that SKI mice displayed elevated lesional necrotic core area in aortic root, which can be relived with the administration of metformin. It's worth noting that SKI mice develop pronounced thinner fibrous cap, which was relieved with the metformin *in vivo* intervention.

### Drp1 suppression stimulates AMPK and attenuates SERCA2 dysfunction directed mitochondrial injury

**Figure 8:**
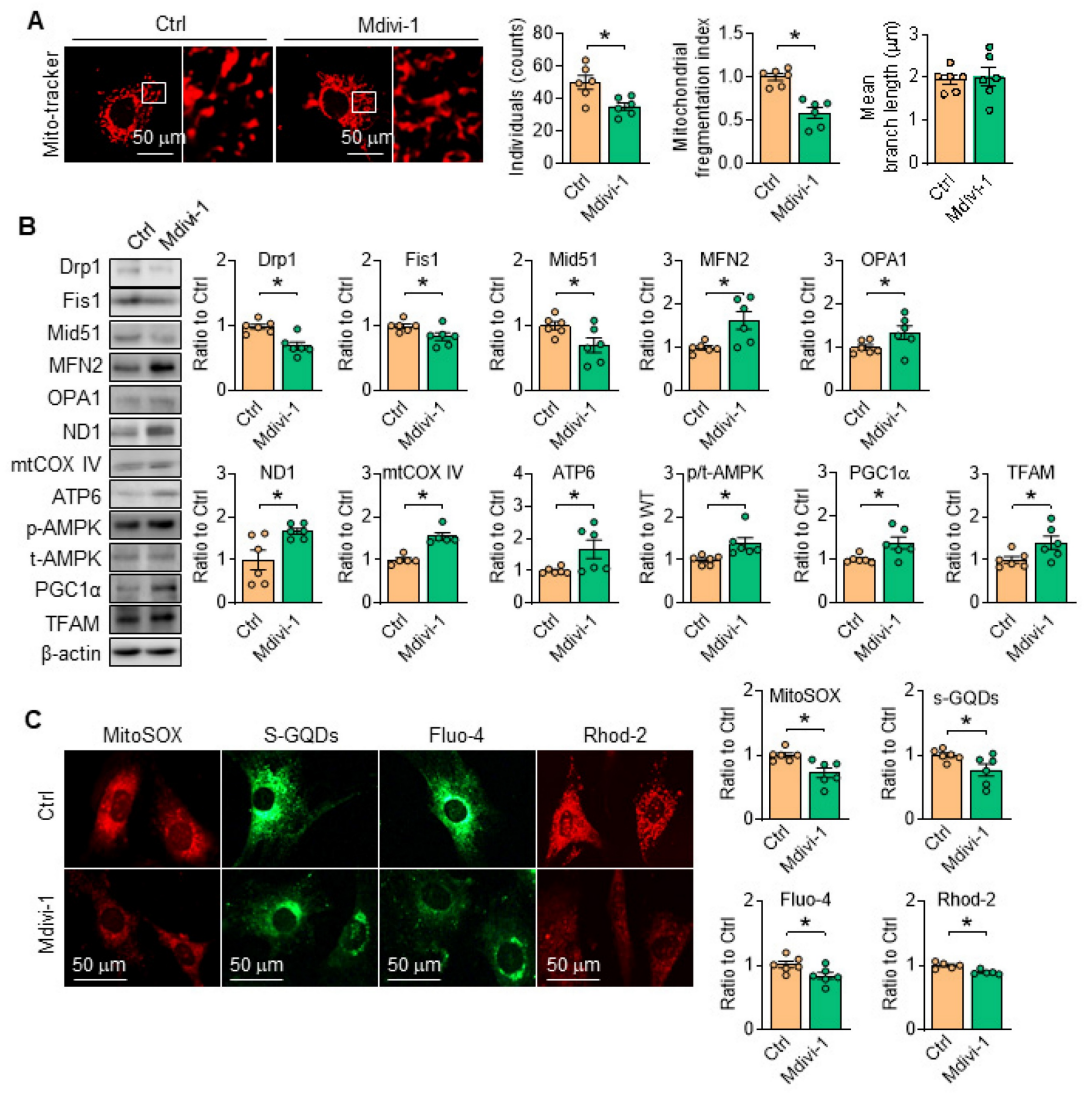
Inhibition of Drp1 by Mdivi-1 suppresses SERCA2 dysfunction-directed mitochondrial fragmentation and restores mitochondrial homeostasis. (A) Representative images and associated quantification of mitochondrial morphology in SKI SMCs with the treatment of Mdivi-1 or its solvent control (Ctrl). (B) Representative western blots of proteins related with mitochondrial fission and fusion (Drp1, Fis1, Mid51, OPA1 and MFN2), oxidative phosphorylation (ND1, mtCOX IV and ATP6), mitochondrial biogenesis (PGC1α and TFAM) and AMPK/ACC1 in SKI SMCs and quantification of band intensities. (C) Representative images and associated quantification of the fluorescence of MitoSOX, s-GQDs, Fluo-4 and Rhod-2 in SKI SMCs with the treatment of Mdivi-1 or its solvent control (Ctrl). *p < 0.05 Mdivi-1 *vs*. Ctrl, n = 5-6 in each group. Data represent the mean of experiments±SEM, unpaired Student *t*-test.

Considering the pivotal role of Drp1 toward mitochondrial dynamics and cell function, the inhibitor of Drp1, Mdivi-1 28, 29 was employed, and the results showed that Mdivi-1 administration *in vitro* relieved mitochondrial fragmentation by modulating the protein levels related with mitochondria fission/fusion (Figure 8A-B), reduced the contents of mROS and promoted ATP production (Figure 8C). Moreover, the protein level of ETC related proteinswere all corrected with Drp1 inhibition (Figure 8B). Additionally, in SKI SMCs with the treatment of Mdivi-1, the contents of cytosolic and mitochondrial Ca^2+^ were both reduced (Figure 8C). Interestingly, AMPK activity was also evidently stimulated with Mdivi-1 administration (Figure 8B), indicating the mutual regulation of AMPK and Drp1. All of the observations indicated that inhibiting mitochondrial division by Drp1 blockade can preserve mitochondrial homeostasis and maintain SMCs function.

### Drp1 suppression by Mdivi-1 ameliorates SERCA2 dysfunction induced atherosclerosis

**Figure 9:**
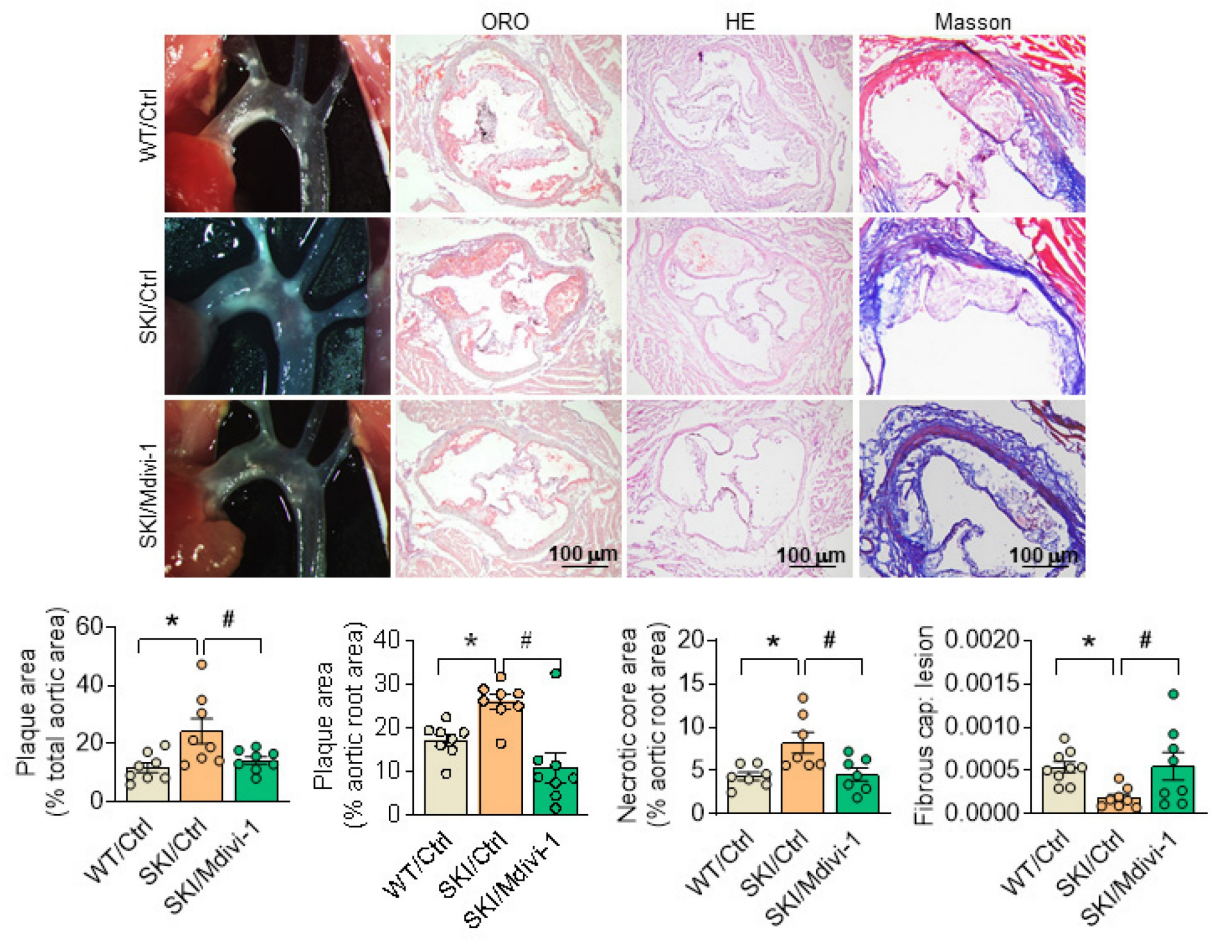
Inhibition of Drp1 by Mdivi-1 alleviates atherosclerosis in SKI mice. Representative images of aortic arch, lesions stained with oil red O, necrosis stained with H&E and collagen deposition stained with Masson in aortic root (up panel) and the quantification in the graph (down panel). *p < 0.05 SKI/Ctrl vs. WT/Ctrl; #*p* < .05 SKI/Mdivi-1 vs. SKI/Ctrl. n = 8. Data represents the mean of experiments±SEM, One-way ANOVA with Tukey's multiple comparisons test.

To further illustrate the role of mitochondrial morphology defects on SERCA2 dysfunction directed atherosclerosis, SKI and WT mice in *ldlr*^-/-^ background were placed on a western diet for 8 weeks, and Mdivi-1 was employed simultaneously. Since the preliminary experimental results showed that Mdivi-1 treatment had almost no effect on WT mice, we didn't focus on *in vivo* intervention in WT mice and only compared three groups of WT/Ctrl, SKI/Ctrl, and SKI/Midivi-1. The atherosclerotic lesions in aortic arch and aortic root, as well as the lesional necrotic core area were all evidently reduced, while thickness of fibrous cap was increased significantly in SKI mice with the administration of Mdivi-1, while no pronounced changes were observed in WT mice (Figure 9).

### Inhibition of Drp1 inhibits SERCA2 impaired directed SMC apoptosis and rescues SKI SMC function

**Figure 10:**
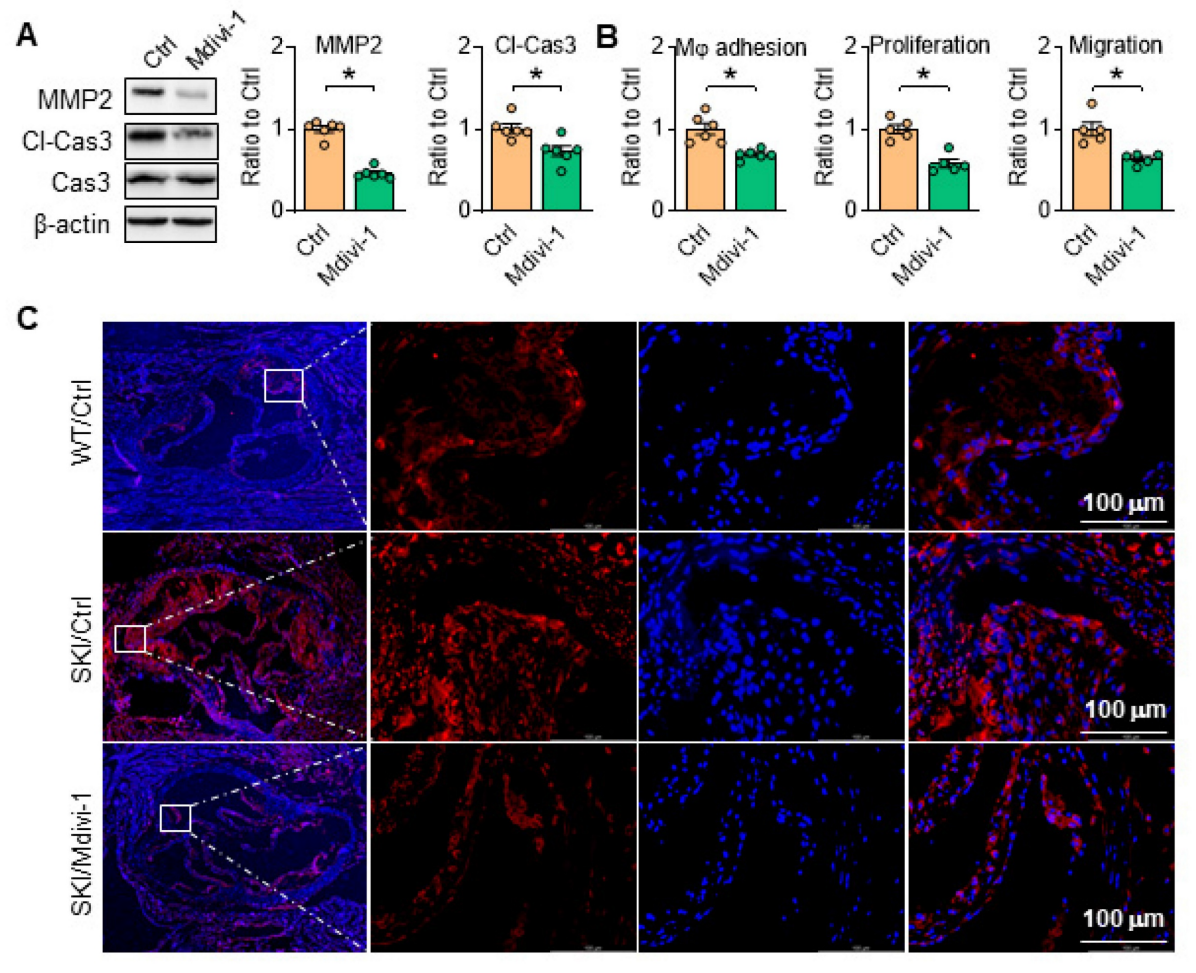
Inhibition of Drp1 inhibits SMC apoptosis and rescues SMC function. Drp1 suppression reduces (A) Representative western blots of MMP2 and cl-Cas3 in SKI SMCs with or without pretreatment of Mdivi-1. (B) The quantitative analysis macrophage adhesion, proliferation and migration of SKI SMCs with or without treatment of Mdivi-1. *p < 0.05 Mdivi-1 *vs*. Ctrl, n = 5-6 in each group. Data represent the mean of experiments±SEM, unpaired Student *t*-test. (C) Caspase 3 (Red) and DAPI (blue) double staining of aortic root atherosclerotic plaques from WT and SKI mice treated with Mdivi-1.

Aortic SMCs can secrete MMP2 to degrade extracellular matrix, and high level of MMP2 can induce apoptosis of SMCs, further making the fibrous cap more susceptible to rupture. As shown in Figure 10A, Mdivi-1 administration blocked MMP2 expression and caspase 3 cleavage, and thus inhibited macrophage adhesion, SMC proliferation and migration in SKI aortic SMCs (Figure 10B). Consistently, the amount of caspase 3 was remarkably increased in aortic root lesions of SKI mice after 10 weeks HFD feeding, which was significantly decreased in corresponding Mdivi-1 treated group (Figure 10C). These observations indicated that Drp1 directed mitochondrial homeostasis plays an important role in SMC apoptotic pathway and the atherosclerotic plaque vulnerability.

## Discussion and Conclusion

Atherosclerosis is a significant global health issue and the primary pathological basis for nearly all cardiovascular diseases. The pathogenesis of atherosclerosis involves complex signaling pathways and interactions among multiple cell types, which remain incompletely understood. Abnormal Ca^2+^ signaling is considered as a crucial regulatory factor 17, 18. Sequestration of Ca^2+^ by SERCA2 is essential to mediates smooth muscle relaxation, and participates many cellular processes, including apoptosis and proliferation. SERCA2 exists in two main isoforms, SERCA2a and SERCA2b, which have distinct roles in calcium regulation. SERCA2a is predominantly expressed in muscle cells and is responsible for Ca^2+^ regulation in SR 30, whereas SERCA2b is housekeeping gene and involved in regulating ER Ca^2+^ level in non-muscle cells 31. We have previously proved that the sulfhydryl group of C674 is a key glutathionylation site to regulate SERCA2 activity, which is easily irreversibly oxidized with prolonged exposure to high levels of ROS/RNS leads, leading to impairment of SERCA2 acitivity 6, 8, 32. In this work, heterozygous SERCA2 C674S knock-in mice in which half of the C674 was substituted by serine674 (S674) were used to mimic the partial irreversible oxidation of C674 under atherogenic conditions, we firstly explored that the dysregulated intracellular Ca^2+^ caused by SERCA2 dysfunction induced AMPK/Drp1 pathway defects, disrupted mitochondrial homeostasis in aortic SMCs, and interfered SMC functions, which aggravated atherosclerosis. Of note, the Pharmacological targeted to AMPK by metformin or Drp1 with Mdivi-1 could both ameliorate SERCA2 dysfunction induced atherosclerosis. Specifically, AMPK stimulation and Drp1 suppression can promote plaque stability by reducing the amount of MMP2 and caspase 3 cleavage, indicating their great potential as therapeutic targets in the treatment of atherosclerosis and in addressing the plaque vulnerability.

AMPK is the central regulator of cellular and organismal material and metabolism in eukaryotes 21, 33. In general, under condition of hypoxia, ischemia, or nutrient deprivation, AMPK can be activated by its upstream kinases such as the tumor suppressor LKB1 and Ca^2+^/calmodulin-dependent protein kinase kinase in the case of aberrant Ca^2+^ changes 34, 35. Conversely, in the present study, AMPK was found to be inactivated in spite of ATP depletion in SKI SMCs. It is hypothesized that t complex regulatory networks may counterbalance the effects of different metabolic pathways. In some cases, elevated cytosolic Ca^2+^ levels might activate energetically expensive pathways, leading to a compensatory decrease of AMPK activity to prevent excessive energy expenditure. Moreover, the interplay between ROS and AMPK activity is complex and reciprocal, with the underlying mechanism being partially understood and a matter of debate 36-39. In this work, increased mROS was observed in SKI SMCs, potentially leading to further DNA oxidative damage (Figure 3A-B). Although the impact of excessive mROS on AMPK activity was not tested in this work, AMPK stimulation significantly reduced mROS level (Figure 5). In another work, we used ROS scavenger Tempol to reduce ROS in SKI mice and found that Tempol could evidently reduce atherosclerotic plagues in the aortic root. This line of evidence all suggests that intracellular Ca^2+^ disturbance caused by SERCA2 dysfunction is pivotal for AMPK pathway defects and mitochondrial disorders, forming a vicious cycle of oxidative stress 40.

Mitochondria, critical integrators of energy production, calcium homeostasis, redox balance and apoptosis, are interconnected and regulated in a coordinated manner. Aberrant of any of these areas can disrupt coordinated regulation and lead to mitochondrial impairment. We found elevated mitochondrial Ca^2+^ in SKI SMCs, which participate the regulation of mitochondrial metabolism, particularly through the regulation of key enzymes in the Krebs cycle 41. Recent work has highlighted the importance of mitochondrial dynamics, particularly the balance between fission and fusion, for mitochondrial homeostasis 42. Usually, mitochondrial fusion seems to be beneficial through distributing proteins, DNA or metabolites, while excessive mitochondrial fission may lead to the accumulation of fragmented mitochondria with impaired ETC and increased mROS in mammalian cells 43-45. In this work, SKI SMCs demonstrated significant mitochondrial disorder and impaired SMC functions. AMPK stimulating reduced mitochondrial fission and promoted mitochondrial fusion by inhibiting the activity of Drp1. This process helps to reduce the mitochondrial fragments, maintain the normal morphology and restore the function of mitochondria as well SMCs. It's worth noting that AICAR treatment in SKI SMCs has no effect on the protein levels of Drp1 and Fis1, but significantly reduced mitochondrial fragmentation. We speculated that on one hand, AICAR treatment significantly upregulated OPA1 and MFN2, which could contribute to the overall reduction in mitochondrial fragmentation. On the other hand, AMPK activation using AICAR also alleviates oxidative stress and restores intracellular Ca²⁺ homeostasis, which thus creates a cellular environment that supports healthier mitochondrial dynamics.

In the context of atherosclerosis, Mdivi-1 has been shown to alleviate atherosclerosis by modulating M1 macrophage polarization 46. In this study, elevated Drp1 level and increased mitochondrial fragmentation were found in SKI SMCs. Drp1 inhibition by Mdivi-1 significantly reduced excessive mitochondrial fragment, sustained mitochondrial homeostasis, and improved SMC function, which also reduced the formation of plaques and the proportion of necrotic cores in SKI mice.

Despite decades of research, our understanding of the mechanisms controlling plaque stability and the risk of plaque rupture during myocardial infarction or stroke remains limited. 47, 48. SMCs form the medial layer of arteries and are the crucial in separating the lipid-rich core from the blood flow, producing and maintaining extracellular matrix components strengthen the cap and prevent rupture 26, 49. We have previously reported that SERCA2 dysfunction promote the shifting of SMCs from a contractile to a synthetic phenotype, accompanied with increased MMP2 and SMCs apoptosis 6, 27. AMPKα2 deficiency has been reported to induce SMC phenotypic switching and exacerbate atherosclerotic plaque instability in a nuclear factor-κB/KLF4 dependent manner 50. However, the effect of Drp1 on atherosclerotic plaque stability has not been extensively studied. Complementary to this, we found AMPK stimulation can also improve plaque stability by rescuing mitochondrial homeostasis and inhibition SMC apoptosis. Particularly critical is that pharmacological intervention by targeting Drp1 apparently increased the thickness of fibrous cap, accompanied with the reduced expression of caspase 3 in the atherosclerotic plaques of aortic root and downregulated of MMP2 and caspase 3 cleavage in SKI SMCs. These observations all indicates the great potential of AMPK or Drp1 as the effective therapeutic targets for atherosclerosis, especially to promote the stability of plaques.

In conclusion, we defined for the first time the contribution of SERCA2 dysfunction induced Ca^2+^ disorders in SMCs to atherosclerosis progression and the formation of vulnerable atherosclerotic plaque. SERCA2 dysfunction causes Ca^2+^ aggregation and mitochondrial Ca^2+^ overloading, drives crosstalk of AMPK and Drp1, which are critical for maintaining mitochondrial homeostasis and SMC function, and thereby accelerates atherosclerosis. Importantly, AMPK/Drp1 pathway defects largely contributes to destruction of atherosclerotic stability, and activating AMPK or inhibiting mitochondrial division are both promising strategies for SERCA2 dysfunction induced atherosclerosis and plaque vulnerability.

## Supplementary Material

Supplementary figure.

## Abbreviations

AICAR: 5-aminoimidazole-4-carboxamide-1-β-D-ribofuranoside
AMPK: AMP-activated protein kinase
ATP6: ATP synthase subunit 6
BMDMs: bone marrow-derived macrophages
C674: cysteine 674
Cl-Cas3: cleaved caspase 3
Ctrl: control
DAPI: 4′,6-diamidino-2-phenylindole
Drp1: dynamin related protein 1
ECs: endothelial cells
ER: endoplasmic reticulum
Fis1: fission 1
H&E: hematoxy-lin/eosin
Mφ: macrophages
Mdivi-1: Mitochondrial division inhibitor 1
Met: metformin
MFI: mean fluorescence intensity
MFN2: mitofusin 2
Mid51: mitochondrial elongation factors 51
MMP2: matrix metalloproteinase
mtCOX: mitochondrial cytochrome-c oxidase subunit
ND1: NADH dehydrogenase subunit 1
OPA1: optic atrophy 1
p-AMPK: phospho-AMPK alpha (Thr172)
PGC1α: peroxisome proliferator-activated receptor-gamma coactivator 1 alpha
ROS: reactive oxygen species
S674: serine 674
SERCA2: sarcoplasmic/endoplasmic reticulum calcium ATPase 2
SKI: SERCA2 C674S knock-in
SMCs: smooth muscle cells
SR: sarcoplasmic reticulum
TFAM: mitochondrial transcription factor A
WT: wild type
