# Supplementary material for: Calcium dysregulation disrupts mitochondrial homeostasis by interfering AMPK/Drp1 pathway to aggravate plaque progression and instability
Source: Theranostics. 2025 Jun 23;15(15):7567–83. doi: 10.7150/thno.112041 (PMC12315820; doi:10.7150/thno.112041)
Supplement: Supplementary file 1 — Supplementary figure. [file thnov15p7567s1.pdf]

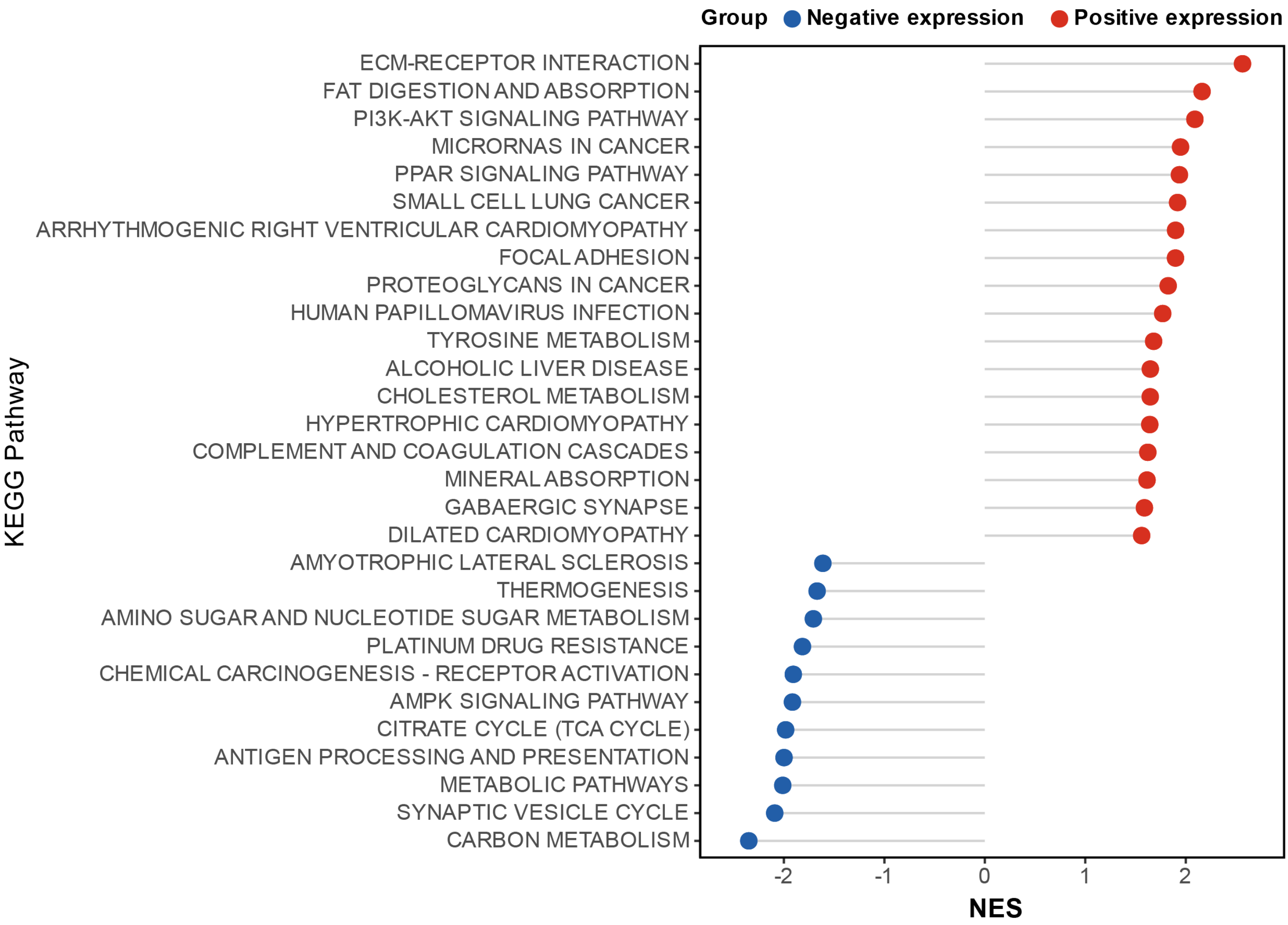

GSEA KEGG enrichment showing all of the changed pathways based on the differential expression of signature proteins.

Supplementary Figure 1
